# Increasing GLP-1 Circulating Levels by Bariatric Surgery or by GLP-1 Receptor Agonists Therapy: Why Are the Clinical Consequences so Different?

**DOI:** 10.1155/2016/5908656

**Published:** 2016-06-12

**Authors:** Chloé Amouyal, Fabrizio Andreelli

**Affiliations:** ^1^Cardiometabolism and Nutrition Institute (ICAN), Heart and Metabolism Department, Pitié-Salpêtrière Hospital (APHP), 75013 Paris, France; ^2^INSERM UMRS U1166 (Eq 6) Nutriomics, UPMC, Pierre et Marie Curie Faculty Paris 6, Sorbonne University, 75013 Paris, France

## Abstract

The “incretin effect” is used to describe the observation that more insulin is secreted after the oral administration of glucose compared to that after the intravenous administration of the same amount of glucose. During the absorption of meals, the gut is thought to regulate insulin secretion by secreting a specific factor that targets pancreatic beta cells. Additional research confirmed this hypothesis with the discovery of two hormones called incretins: gastric inhibitory peptide (GIP) and glucagon-like peptide 1 (GLP-1). During meals, specific cells in the gut (L and K enteroendocrine cells) secrete incretins, causing an increase in the blood concentrations of, respectively, GLP-1 and GIP. Bariatric surgery is now proposed during the therapeutic management of type 2 diabetes in obese or overweight populations. It has been hypothesized that restoration of endogenous GLP-1 secretion after the surgery may contribute to the postsurgical resolution of diabetes. In 2005, the commercialization of GLP-1 receptor agonists gave the possibility to test this hypothesis. A few years later, it is now accepted that GLP-1 receptor agonists and bariatric surgery differently improve type 2 diabetes. These differences between endogenous and exogenous GLP-1 on glucose homeostasis emphasized the dual properties of GLP-1 as a peptide hormone and as a neurotransmitter.

## 1. Introduction

Since the 1960s, when the effect of incretin was discovered, there has been a considerable amount of work on the physiology of incretins (the best known being glucagon-like peptide 1 [GLP-1]) and their mode of action. Progress has been made, but much remains unknown about the mechanism of action of incretin, despite the existence of several pharmacological compounds altering the abundance or half-life of GLP-1, which are currently used as hypoglycemic agents. These issues, and the potential role of GLP-1 in the remission of type 2 diabetes observed after bariatric surgery, are important. The almost ubiquitous presence of the GLP-1 receptor contrasts with the extremely short half-life of the hormone in the circulation (approximately one minute) due to its rapid degradation by the endothelial enzyme dipeptidyl peptidase IV (DPP-IV). The recent discovery of a lymphatic transport system for GLP-1 (DPP-IV is absent from this pathway) and the probable activity of GLP-1 as a neurotransmitter raise questions about the exact role of this incretin in the physiology of the whole organism. Below, we consider the bases for conventionally accepted ideas and interpretations and how they can also lead to new hypotheses.

## 2. Cells That Secrete GLP-1

GLP-1 is secreted by intestinal L cells, which are very rare and highly specialized enteroendocrine cells of the intestine. Indeed, enteroendocrine cells make up only 1% of intestinal cells, and only a subset of this physiologically important family secrete GLP-1. These cells are distinguished by a characteristic tapered shape: the narrow end of the cell is in contact with the intestinal lumen and the wide end contains secretory granules [1]. The rarity of these cells makes it technically difficult to quantify them or to study their distribution throughout the gastrointestinal tract. It is generally accepted that the L cells are present in the last segment of the small intestine and colon [2]. However, recent data show that the reality is more complex, because L cells are present also in the duodenum in humans [3]. Thus, L cells are probably distributed throughout the length of the intestinal tract. The abundance of these cells may progressively increase from the duodenum to the colon, although the existence of such a gradient shown in mice and in pigs [4] has not been formally demonstrated in humans.

Another peculiarity of L cells discussed in the literature is that some of them produce only GLP-1, others produce only GIP (another incretin typically secreted by duodenum K cells), and some cells produce both incretins [3]. It is still unclear whether these cell markers on stained sections of intestine reflect different patterns of secretion* in vivo* and whether specific mechanisms regulate secretion by these cell types. Importantly, it is difficult to explain the quick and parallel increase in GIP and GLP-1 secretions after a meal if we consider the classical localization of GIP cells (proximal gut) and of GLP-1 cells (distal gut) [5]. In this context, the demonstration by immunohistochemical studies that cosecreting cells (GLP-1/GIP cells) are present in the proximal gut would help explain this controversy [3, 6, 7]. Nevertheless, recently, Svendsen et al., challenged the concept of GLP-1/GIP cosecreting cells using transgenic mice in which GIP and GLP-1 cells express different fluorescent markers [8]. By combining results obtained from primary cultures of the upper small intestine, single GLP-1 and GIP-producing cells, and isolated perfused proximal mouse small intestine, the authors demonstrated that cells secreting both incretins are rare (around 5%) in upper small intestinal cell suspensions. In addition, GIP and GLP-1 secretions were differentially regulated. If glucose, KCL, and forskolin +IBMX enhanced secretion of both GIP and GLP-1 from baseline, GLP-1 secretion was enhanced only by bombesin/neuromedin C. Interestingly, bombesin 2 receptor expression has been found only in GLP-1 positive cells and not in GIP positive cells suggesting that L and K cells are different in terms of functionality and expression of cell markers. Thus, the conclusion of this study was that GLP-1 cells and GIP cells of the proximal gut of mice have to be considered as distinct cells and that colocalization of both hormones does not always result in cosecretion of both incretins [8]. Additional studies focused on the origin and differentiation of cells secreting GLP-1. Original results about expression of peptide precursor in intestinal cells have been obtained in a model of transgenic mice expressing green fluorescent protein (eGFP) under the control of the CCK promoter [9]. In this model, half of CCK-eGFP positive cells expressed another peptide than CCK, including glucagon-like peptide 1 (GLP-1), gastric inhibitory peptide (GIP), peptide YY (PYY), neurotensin, and secretin, while a smaller fraction of CCK-eGFP positive cells expressed two peptide precursors in addition of CCK. In a following step, potential functional consequences of coexpression of different gut hormones in the intestinal-specific lineage of enteroendocrine cells were analyzed. Importantly, Grunddal et al. showed that GLP-1 cells in the crypts coexpressed PYY and neurotensin as they move up the villus suggesting that these cells acquired additional functionalities during their migration [10]. The three peptides were cosecreted from both perfused small intestines and colonic crypt cultures and participated together to the regulation of food intake, gastric emptying, and glucose homeostasis demonstrating that cosecretion of gut hormones has functional and metabolic consequences [10]. Little is known about the mechanisms that participated in the development of intestinal-specific lineage of enteroendocrine cells. It was recently demonstrated that miR-375 negatively regulates the development of intestinal enteroendocrine cells but further studies are needed to provide more information on this topic [11].

## 3. GLP-1 Secretion

Determining the distribution of cells in the gut is not a trivial problem. The secretion of GLP-1 is thought to depend on the detection of substrates such as glucose, fats, and amino acids in the digestive lumen, either alone or in combination following a meal [12]. Glucose strongly stimulates the secretion of GLP-1 and has been the most extensively studied substrate. It is unclear whether glucose directly or indirectly stimulates the secretion of GLP-1. Indeed, in healthy individuals, the oral ingestion of glucose strongly stimulates the secretion of both GLP-1 and insulin, whereas the intraduodenal administration of glucose has little effect on GLP-1 and no effect on insulin secretion (see below) [12]. This suggests that the presence of glucose in the intestinal lumen* in vivo* is probably not in itself sufficient to stimulate GLP-1 secretion.

However, cells lines, including GLUTag, STC-1, and NCI-H716, secrete GLP-1 in response to glucose* in vitro*, suggesting a direct effect of this substrate [13–15]. In fact, the direct effect of glucose on the secretion of GLP-1 in these* in vitro* models is itself dependent on an increase in the concentration of Ca^2+^ in the cytoplasm, which may occur by its release from intracellular stocks or by Ca^2^ influx across the plasma membrane, presumably following membrane depolarization. Other mechanisms have also been implicated in the secretion of GLP-1. GLUTag cells secrete GLP-1 when exposed to tolbutamide, which closes an ATP-dependent K^+^ channel leading to membrane depolarization, the opening of voltage-dependent calcium channels, and the intracellular influx calcium [16, 17]. Therefore, these cells behave like pancreatic *β*-cells* in vitro*. In clinical practice, things are less clear. Indeed, sulfonylureas, which are widely used to treat diabetes, do not stimulate the secretion of GLP-1* in vivo* despite the presence of K^+^ channels on human L cells [18]. Another partner that may be necessary for the secretion of GLP-1 is the SGLT-1 transporter, which is the major glucose transporter on the intestinal luminal side. Indeed, the activity of SGLT-1 in GLUTag cells is associated with the secretion of GLP-1 [18]. In animals, the activation of SGLT-1 is required for GLP-1 secretion stimulated by D-glucose, D-galactose, methyl alpha-D-glucoside, and 3-O-methyl-D-glucose [19]. Moreover, 3-O-methyl-D-glucose is not metabolized by intestinal cells, suggesting that the compound itself, and not any metabolite produced from it, is important for the secretion of GLP-1. D-Fructose stimulates GLP-1 secretion by a mechanism that does not involve the transport of sodium. N-Acetyl-D-glucosamine and 2-deoxy-D-glucose, which are not substrates of SGLT-1 or the fructose transporter, do not modify the secretion of GLP-1 [19].

Cells secreting GLP-1 express specific receptors on their luminal surface that are also found on the tongue where they are involved in taste. These taste receptors (in particular T1R and T2R) are thus expressed both in the tongue and at the luminal end of L cells secreting GLP-1 [20]. They are coupled to intracellular signaling, through a particular G protein, called gustducin, which was discovered in 1992 [21]. This pathway involves phospholipase C, which signals via inositol-3-phosphate and diacylglycerol, resulting in the mobilization of intracellular calcium stores and finally the exocytosis of vesicles containing GLP-1 at the basal end of the cell. Mice deficient for either T1R3 or gustducin do not secrete GLP-1* in vivo* when given glucose [21]. These findings suggest a model in which the secretion of GLP-1 depends on the detection of substrates by cellular receptors of the digestive system, such as T1R and T2R, which are present on L cells [16]. As recently suggested, taste receptors expressed in the oral cavity and the gastrointestinal tract could play an important role not only in taste sensation but also in hormonal responses to nutrient load [22]. The interesting paper of Hsueh et al. showed evidence that taste receptor dysfunction in humans could contribute to glucose homeostasis alterations. In a candidate gene study within the Amish Family Diabetes Study (AFDS), the authors demonstrated that haplotype of a subtype of taste receptor (TAS2R9) is associated with altered glucose and insulin homeostasis [23]. Importantly, TAS2R9 is expressed in enteroendocrine cells and functional analysis showed that one TAS2R9 polymorphism (rs3741845 SNP) reduced the capacity of some ligands to bind the extracellular loop of this receptor with subsequent alteration of enteroendocrine secretions. As a consequence, polymorphisms in taste receptors could alter incretin effect which in turn may increase diabetes risk.

## 4. Why Is It So Different to Increase GLP-1 Levels by GLP-1 Receptor Analogs Therapy or by Bariatric Surgery?

Bariatric surgery is increasingly being proposed to treat obesity following the failure of medical management and in cases of morbid obesity, or obesity with one or more comorbidities. There are several operative methods, and the two main techniques currently used worldwide are the Sleeve gastrectomy and the Roux-en-Y gastric bypass surgery. Although the success of these techniques, concerning both food intake and the resolution of type 2 diabetes, depends on several factors, many studies have suggested an important role for postoperative concentrations of endogenous GLP-1 for the remission of type 2 diabetes [24]. Indeed, obesity and type 2 diabetes impair the secretion of GLP-1 during meals; and bariatric surgery techniques favor the correction of this secretory defect. GLP-1 acts as a satiety factor and positively influences glucose metabolism; therefore, it is tempting to correlate the beneficial effects of surgery with a postoperative increase in GLP-1 circulating levels [25]. Nevertheless, the assumption that GLP-1 secretion plays a key role in the remission of type 2 diabetes after the gastric bypass surgery is now challenged, particularly by the intriguing observation that GLP-1 analogs are not able to promote remission of type 2 diabetes as surgical procedures do. GLP-1 analogs are effective agents for the treatment of type 2 diabetes with many advantages such as body weight loss during therapy [26, 27] and it has been postulated that GLP-1 analogs could be considered, at least in part, as bariatric surgery “mimetics” [24, 25]. This is not exactly the case. The discrepancy between bariatric surgery and intensive medical therapy of type 2 diabetes including GLP-1 analogs for the control of glucose homeostasis is probably not related to differences in plasma concentrations of GLP-1 obtained by the two approaches. Indeed, gastric bypass surgery results in physiological concentrations of active GLP-1 (with a peak of about 20 pmol/L 20 minutes after a meal before to come back to the basal value), whereas GLP-1 receptor agonists can produce higher concentrations of active GLP-1, reaching a stable plateau in the range of 60–70 pmol/L (thus circulating levels of active GLP-1 are higher than those obtained after a bariatric surgery) [28]. Why can we explain that, during GLP-1 analogs therapy, supraphysiological GLP-1 circulating levels are not sufficient to promote type 2 diabetes resolution? Why restoration of physiological levels of GLP-1 secretion is sufficient for the remission of diabetes after bariatric surgery? Different hypotheses can be analyzed. First, administration of GLP-1 receptor agonists may not have the same effects as bariatric surgery because the effects of surgical procedures are not limited to GLP-1. Indeed, the known mechanisms of the beneficial effects of bariatric surgery on diabetes are complex and interconnected and include (this list is not exclusive) changes in diet and behaviour, as well as changes in hormones secretion (insulin, leptin, ghrelin, adiponectin, peptide YY, and glucagon), bile acid flow, intestinal gluconeogenesis, low grade inflammation, and gut microbiota [29]. A second important hypothesis to be discussed is that GLP-1 is not essential for the resolution of diabetes after bariatric surgery [30]. This has been suggested by the observation that an intraperitoneal infusion of exendin [9–39] amide (a specific GLP-1 antagonist without any agonistic properties) [31] failed to abrogate the improvement of glucose homeostasis in a murine model of bariatric surgery and also in humans [30, 32]. In addition, even if glucose metabolism of rodents and humans is different, it is interesting to notice that the metabolic effects of the Roux-en-Y gastric bypass or of the vertical Sleeve gastrectomy procedures are preserved in rodents deleted for the GLP-1 receptor [33, 34]. Lastly, differences between GLP-1 circulating levels after bariatric surgery or GLP-1 analogs therapy raise also the question of the importance of the circulating concentration of active GLP-1 [28]. Is circulating GLP-1 really responsible for its metabolic effects? Is detection of GLP-1 in gut (as restored by bariatric surgery procedures) the key mechanism of action of this hormone?

## 5. Mode of Action of GLP-1: The Classical Pathway

GLP-1 meets all of the criteria for a hormone according to the definition proposed by Starling in 1905 [35]. GLP-1 is secreted by specialized cells and is released into the bloodstream where it is delivered to distant target tissues possessing receptors specialized for this molecule. Its binding to its receptor brings about a specific response in the target tissue. The GLP-1 receptor is a G protein-coupled receptor that was cloned in 1992 from pancreatic cells by Thorens [36]. Many peripheral tissues possess GLP-1 receptors similar to those on pancreatic *β*-cells [37]. These tissues are thus potential targets of GLP-1 and include the brain (especially the paraventricular and arcuate hypothalamic nuclei, as well as the area postrema, nucleus of the solitary tract, and dorsal motor nucleus of the vagus in nonhuman primate and in mice) [38], kidneys and lungs (smooth muscle cells in the walls of arteries and arterioles), the digestive tract (the duodenum, the parietal cells and smooth muscle cells in the muscularis externa of the stomach, and the myenteric plexus neurons throughout the gut), the heart (myocytes of the sinoatrial node) [39], and possibly pancreatic *α*-cells [37]. Thus, GLP-1 receptors are abundant in many tissues, but GLP-1 itself is rapidly degraded in the circulation. GLP-1 is degraded by an enzyme present on the endothelium (DPP-IV) and the half-life of active GLP-1 in the blood stream is very short (less than 1 minute). In studies involving the catheterization of different body compartments in pigs, Holst estimated that less than 10% of active GLP-1 secreted into the digestive tract can be found intact in the bloodstream and hence reach and activate specific receptors on tissues via endocrine signaling (or classic hormonal signaling) [37]. This strong, permanent inactivation process is unusual among hormones and its purpose or function remains to be discovered.

## 6. Mode of Action of GLP-1: Alternative Pathways

In 2007, D'Alessio and colleagues showed that a significant proportion of GLP-1 secreted into the digestive tract in rats is recovered by the lymphatic system and the thoracic duct (which itself ends in bloodstream at large vessels at the base of the neck) [40]. The concentrations of active GLP-1 in the lymphatic system are 30 times higher than those in peripheral blood (the peak of GLP-1 after caloric oral gavage is 15 pmol/L in peripheral blood and 400 pmol/L in the thoracic duct). One may have expected GLP-1 to accumulate slowly in this compartment, but this is not the case: after a meal, the peak concentration of active GLP-1 is observed in the thoracic duct only 10 minutes after its concentration peaks in the general circulation. Concentrations of active GLP-1 in the two compartments then decay at a similar rate. DPP-IV is not found in the thoracic duct, which probably explains the very high concentrations of active GLP-1 in this compartment [40]. Thus, it is probable that a significant proportion of secreted GLP-1 passes into the thoracic duct, which enables it to enter the circulation without degradation. However, little is known about the physiological significance of this compartment.

## 7. Mode of Action of GLP-1: A Hormone or a Neurotransmitter?

**Figure 1:**
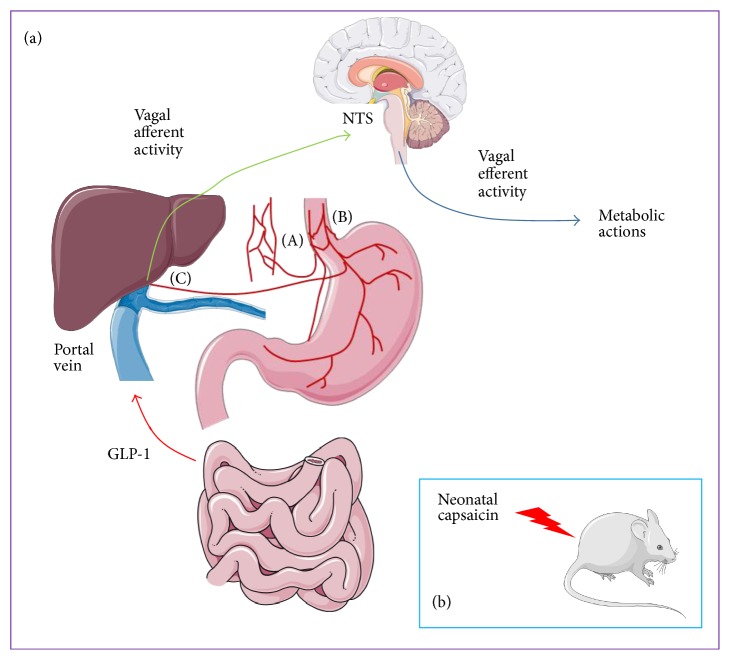
Evidence that physiological metabolic actions of GLP-1 are dependent on integrity of vagal nerve. (a) Stimulation of the celiac branch of the subdiaphragmatic vagus nerve (A) significantly stimulated the secretion of GLP-1 [44]. Enhancement of insulin secretion and reduction of glucagon secretion during OGTT by DPP-IV inhibitor or by GLP-1 analogs were reduced in vagotomized subjects (B) [45, 46]. GLP-1 is secreted during meal by the gut and partly reached the portal vein. Chemical destruction of the afferent fibers of the periportal vagus nerve including the hepatoportal sensor (C) limits the effects of portal GLP-1 on glucose metabolism [50]. Administration of physiological doses of GLP-1 into the portal vein modified the electrical activity of vagus nerve fibers [51] and portal perfusion of low doses of an antagonist of GLP-1 caused hyperglycemia during the concomitant oral administration of glucose [52]. (b) In mice denervated at birth by the injection of capsaicin, incretin effect was lost [43]. NTS: nucleus tractus solitarius. Pictures of organs and mice from Servier Medical Art.

GLP-1 has a special role in the brain. GLP-1 in the blood and thus present as a peripheral hormone is able to reach some structures in the brain due to the permeability of the blood-brain barrier at particular places (such as the arcuate nucleus). However, GLP-1 is also synthesized in the brain and acts as a neurotransmitter [41]. Various peripheral signals (circulating concentrations of substrates or hormones, fasting, and meals) modulate the synthesis and release of GLP-1 in the brain. GLP-1 may also act as a neurotransmitter at a distance. Indeed, shortly after its release, GLP-1 can be detected in the intestine by the vagal afferent nerves [42]. This information is then relayed to the brain, which in turn may act on peripheral tissues through efferent vagal nerves, resulting in GLP-1-mediated effects via the nervous system [37] (Figure 1). This loop enables GLP-1 to act on a target tissue via the vagus nerve, without the need to physically reach the target tissue. This mode of action is consistent with the very limited half-life of the hormone and is made possible by the substantial innervation of the gut. This has been extensively demonstrated in various murine models, including mice denervated at birth following the early postnatal injection of capsaicin (a neurotoxin from the alkaloid family). In this model, physiological doses of GLP-1 do not stimulate the glucose-dependent secretion of insulin, reflecting the need for the integrity of nerve pathways to mediate the effects of exogenous GLP-1 on insulin [43]. Control of GLP-1 secretion by the nervous system is also suggested if we consider that, during meals or following the oral administration of glucose, the secretion of GLP-1 peaks rapidly (usually between 10 and 15 minutes in healthy individuals) which is incompatible with physical contact between food and intestinal L cells [37]. More directly, the stimulation of the celiac branch of the subdiaphragmatic vagus nerve significantly stimulated the secretion of GLP-1 in an* in situ* model of the rat gastrointestinal system [44]. In addition, contribution of the vagal nerve for the major physiological effects of GLP-1 was also demonstrated by the reduction of some effects of GLP-1 and of DPP-IV inhibitors in vagotomized subjects. Indeed, Plamboeck et al. studied the metabolic consequences of exogenous GLP-1 in 20 truncally vagotomized subjects with pyloroplasty and 10 matched healthy controls [45]. All subjects received exogenous GLP-1 (7–36 amide) or saline infusions during and after a standardized liquid mixed meal. Interestingly, exogenous GLP-1 infusion failed to reduce food intake, gastric emptying, and glucagon secretion in truncally vagotomized subjects suggesting that vagal innervation contributed to GLP-1's actions. Contribution of vagal innervation to hypoglycemic effect of DPP-IV inhibitors has also been discussed by the same team. Truncally vagotomized subjects and healthy subjects underwent an oral glucose tolerance test (OGTT) either with or without DPP4 inhibition in randomised order [46]. In a separate occasion, in all subjects, the plasma glucose profile from the OGTT day without DPP4 was duplicated by a glucose infusion at a variable rate (isoglycemic i.v. glucose infusion). During OGTT and glucose infusion, plasma glucose, insulin, C-peptide, GLP-1, glucagon, and GIP were measured. The results showed that isoglycemia was obtained with 25 ± 2* * g glucose in vagotomized subjects and 18 ± 2* * g in controls (*P* < 0.03) suggesting a reduction of the gastrointestinal-mediated glucose disposal in vagotomized compared with the control group. Interestingly, both groups were similar for the incretin effects. Despite this, enhancement of insulin secretion and reduction of glucagon secretion during OGTT by DPP-IV inhibitor were reduced in vagotomized subjects suggesting that some physiological effects of GLP-1 involve vagal transmission. How the vagal innervation contributes to the GLP-1 effects is not completely understood.

Another example of GLP-1 acting through neural pathways is the detection of portal glucose by the “hepatoportal sensor.” It was recently shown that portal glucose plays an important role in regulating the fate of glucose in the rest of the body. Following its ingestion, glucose is sensed in the portal vein by a complex system called the hepatoportal sensor, which is still not fully understood [47]. This system requires the afferent fibers of the vagus nerve and the glucose transporter GLUT2. Intraportal administration of glucose is sufficient to increase the insulin sensitivity of peripheral tissues and to lower blood sugar levels, even if the secretion of insulin is artificially blocked by perfusion with somatostatin [48]. This suggests that an increase in the portal glucose concentration is detected by the sensor and the signal is transmitted by the afferent vagus nerve to the brain which in turn controls the metabolism of peripheral tissues via the nervous system (in particular via the efferent fibers of the vagus nerve). The administration of intraportal glucose has no effect on peripheral tissues in rodent models invalidated for GLUT2 or if the portal fibers of the vagus nerve are destroyed [49].

Following the ingestion of glucose, GLP-1, in addition to glucose itself, appears in the portal circulation. It has been reported that portal glucose has no effect on the metabolism of glucose in mice invalidated for the GLP-1 receptor, showing the importance of GLP-1 in the hepatoportal sensor [50]. In 1996, Nakabayashi and colleagues found that administration of physiological doses of GLP-1 into the portal vein modified the electrical activity of vagus nerve fibers, which suggests the existence of a neurological system for the detection of this hormone [51]. Vahl and colleagues later showed that the portal perfusion of low doses of an antagonist of GLP-1 caused hyperglycemia during the concomitant oral administration of glucose [52]. Furthermore, the intrajugular perfusion of this antagonist had no deleterious effect on glucose tolerance, suggesting that the hepatoportal sensor, via the nervous system, is necessary to mediate the effects of portal GLP-1 on insulin secretion upon the ingestion of glucose (incretin effect). The authors also demonstrated the existence of GLP-1 receptors in portal nerve fibers and the associated ganglia, thereby providing a physical basis for the detection of portal GLP-1. Accordingly, lesions of the ganglia innervating the portal or chemical destruction of the afferent fibers of the periportal vagus nerve limit the effects of portal GLP-1 on glucose metabolism [50]. Thus, it is probable that portal GLP-1, through a specific network of nerves, modifies the activity of several brain structures (particularly brainstem nuclei, the arcuate nucleus, and paraventricular nucleus of the hypothalamus), which are essential for the metabolic actions of this hormone [37].

An essential role for GLP-1 is also to modulate glucagon secretion [53]. Inappropriate glucagon levels in blood are observed in fasting and after a meal in both type 1 and type 2 diabetes patients [54]. Because excess of glucagon availability contributed to increase glucose levels, reduction of glucagon secretion and/or action is now considered as an important therapeutic goal in diabetes [55]. There is a compelling body of evidence to support the assumption that GLP-1 contributes to the regulation of glucagon secretion. In contrast to GIP, which increases glucagon secretion both in controls and in type 2 diabetes subjects, GLP-1 reduces glucagon secretion in addition to increasing insulin secretion [56]. Interestingly, as for insulin secretion, GLP-1's effect on glucagon secretion is also glucose dependent [57]. Interestingly, plasma glucagon concentrations in the fasting state and after a meal increased during GLP-1 receptor antagonist exendin 9–39 administration suggesting that endogenous GLP-1 inhibits glucagon secretion [58, 59]. Is glucagon secretion regulated directly or indirectly by GLP-1? GLP-1 diminished glucagon secretion of isolated perfused porcine pancreas as described for the first time in 1988 suggesting a direct and/or a paracrine effect of GLP-1 on *α*-cells function [60]. Nevertheless, until now, it has been very difficult to obtain convincing data that would support meaningful expression of the GLP-1 receptor in rodent or human *α*-cells [61, 62]. Hence it has been proposed that the effects of GLP-1 to inhibit glucagon secretion are indirect, via either the nervous system, the *β*-cell (through inhibitory factors such as insulin, GABA, or zinc), the *δ*-cell, or additional indirect mechanisms [56]. The observation that GLP-1 receptor agonists reduced glucagon secretion in type 1 diabetes patients (C-peptide negative patients) demonstrated that the inhibitory effects of GLP-1 on *α*-cells is not totally dependent on *β*-cells [63]. As demonstrated in rodents, somatostatin receptor subtype-2 is important for the control of glucagon secretion [64]. Because somatostatin secretion from pancreatic *δ*-cells is stimulated by GLP-1, it could be possible that GLP-1 reduces glucagon secretion in a paracrine manner [65].

## 8. Why so Many Different Modes of Action?

GLP-1 thus has numerous mechanisms of action, and their physiological relevance and importance need to be determined. Does GLP-1 act preferentially as a local neurotransmitter (in the intestine or portal vein) or as a circulating hormone? Are these two major modes of action disrupted in particular pathological contexts? These fundamental issues have been addressed by both physiological studies with GLP-1 receptor agonists and inhibitors of DPP-IV.

First, note that the circulating concentrations of GLP-1 during fasting are low, but not negligible. What is the importance of this basal concentration of GLP-1? Even if it has been controversial, DPP-IV is thought to be permanently active; therefore, GLP-1 must be constantly secreted, and a balance is established between its production and its degradation to ensure significant and stable blood levels in the basal state [66]. However, L cells that secrete GLP-1 (at least* in vitro*) do so following the detection of the appropriate substrates (as summarized above). In resting conditions* in vitro*, GLP-1 is virtually undetectable in these cell systems. However,* in vivo*, in mice, rats, pigs, and humans, basal circulating concentrations of GLP-1 remain stable at concentrations around 5 to 10 pmol/L [37]. One possibility is that this basal secretion provides a reservoir of GLP-1 to enable the progressive release of the hormone. However, this is unlikely because the only known potential “reservoir” of GLP-1 is in the thoracic duct, which does not possess L cells and is clearly supplied by digestive secretions. Returning to portal glucose detection by the hepatoportal sensor, the utilization of peripheral glucose and resulting hypoglycemia are similar following the coperfusion of glucose and GLP-1 into the portal vein or the perfusion of glucose alone. Conversely, the joint perfusion of glucose and a GLP-1 antagonist into the portal vein alters the peripheral utilization of glucose and prevents the onset of the hypoglycemia that occurs without the antagonist [50]. This confirms that portal glucose requires activation of the GLP-1 receptor to initiate a nervous response that will in turn affect the peripheral organs. However, the authors state that this also suggests that basal GLP-1 acts as a sensitizing agent in the hepatoportal sensor, to maximize the detection of portal glucose as soon as it appears and to amplify the resulting signal [50]. Basal concentrations of GLP-1 may thus enable the hepatoportal sensor to be constantly ready (or preconditioned) to detect and to transmit the signal arising from portal glucose as efficiently as possible.

Another way to address these issues is to use mouse models. Is GLP-1 essential for viability? The answer is no. Mice invalidated for GLP-1 receptor do not have any developmental defects and show only minor disturbances of blood sugar levels, notably after the ingestion of glucose, in accordance with the role of GLP-1 as an incretin [67]. In addition, these mice show no appetite abnormalities and do not develop obesity: this suggests that, in physiological conditions, GLP-1 has no major effect on the regulation of food intake, which instead depends on many other systems. In a subsequent article, the same group showed that deletion of the GLP-1 receptor has no effect on insulin sensitivity, the insulin content of pancreatic *β*-cells, or glucagon secretion; instead, the only anomaly observed was the weak secretion of insulin following the ingestion of glucose, which was substantially lower than in control mice [68]. The authors therefore concluded that the only experimentally demonstrated physiological effect of GLP-1 is restricted to its role as an incretin. The same group later published the results of three mouse models (one invalidated for the GLP-1 receptor, another for the GIP receptor, and a third for both receptors) fed either a standard laboratory diet or a high fat diet [69]. Once again, the loss of function of one or both receptors had little influence on the metabolism of glucose. Indeed, only blood sugar levels after the ingestion of glucose are modestly disrupted, and this is due to the weak stimulation of insulin secretion. A high fat diet had little effect on these results: mice invalidated for both the GLP-1 and GIP receptor were even protected from the deleterious effects of fat diet! Insulin sensitivity was also preserved in these models. These observations are important because they strongly suggest that, in physiological situations, GLP-1 has probably only one essential role: the amplification of insulin secretion during food intake. Although mice lacking both the GLP-1 and GIP receptor show modest compensatory anomalies in the development of pancreatic *β*-cells when fed a high fat diet, these studies suggest no physiological role for GLP-1 in the control of insulin sensitivity, glucagon secretion, or food intake in mice. These results contrast with the demonstrated effects of the pharmacological administration of GLP-1 or receptor agonists of GLP-1. The physiological effects of GLP-1 should therefore not be confused with those observed following the pharmacological administration of GLP-1.

Similarly, many reports have suggested that the intracerebral receptor for GLP-1 is essential for its metabolic effects. Indeed, the central administration of a GLP-1 antagonist causes hyperglycemia during glucose tolerance tests, suggesting that central GLP-1 receptors regulate blood sugar levels following the ingestion of glucose [70]. Conversely, the central administration of GLP-1 increases the glucose-stimulated secretion of insulin. In addition, the administration of GLP-1 directly into the arcuate nucleus impairs hepatic glucose production whereas the administration of GLP-1 into the paraventricular nucleus of the hypothalamus reduces food intake. Thus, the action of exogenous GLP-1 perfused directly into the brain depends on structure that receives it and the effects of GLP-1 on satiety are dissociated from its effects on glycemic control. This dissociation is particularly true for the central effects of GLP-1 receptor agonists. The GLP-1 analog, liraglutide, is able to cross the blood-brain barrier [71]. Sisley and coworkers showed that, in a mouse model invalidated for GLP-1 specifically in the brain, peripherally injected liraglutide was still able to induce the incretin effect* in vivo* but had no effect on satiety [72]. Thus, the central GLP-1 receptor appears to be essential for mediating the supraphysiological effects of GLP-1 on food intake but does not appear to be involved (or plays only a minor role) in the incretin effect. It is also possible that some of the effects of the analog involve other pathways besides those targeted by GLP-1 itself, that is, pathways specific to the analog that remain to be discovered. Again, it is important to emphasize that these conclusions involve receptor agonists of GLP-1 and not physiological conditions, because the invalidation of GLP-1 specifically in neurons has no deleterious effect on the regulation of blood sugar and food intake [72]. These data emphasize that the physiological effects of GLP-1 (restricted to the incretin effect) should not be confused with the effects of GLP-1 receptor agonists, of which the two main effects involve appetite (which requires the presence of central GLP-1 receptors) and improvement of glucose tolerance (which is less dependent on central GLP-1 receptors).

**Figure 2:**
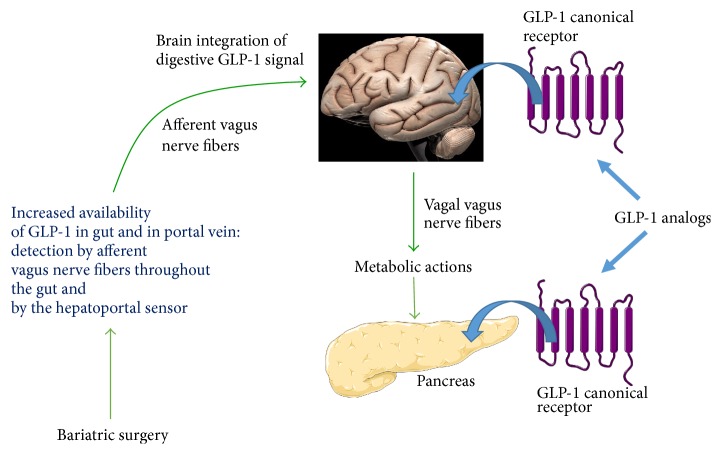
Differences on GLP-1 secretion and action between bariatric surgery and GLP-1 analogs. In contrast to GLP-1 analogs that targeted directly GLP-1 canonical receptor in peripheral tissues (brain and pancreas as examples), bariatric surgery increased GLP-1 secretion by the gut. Then, GLP-1 is detected by afferent vagus nerve fibers throughout the gut and in the portal vein. This allowed brain integration of intestinal GLP-1 secretion and consequently coordinated metabolic actions in peripheral tissues by efferent vagus nerve fibers. Pictures of organs from Servier Medical Art.

The same confusion may exist between the restoration of physiological concentrations of GLP-1 after gastric bypass surgery and the extrapolation of data obtained with high concentrations of GLP-1 receptor agonists, which cannot mimic the effects of gastric bypass surgery on diabetes and satiety. It is now clear that GLP-1 itself cannot account for all the beneficial effects of gastric bypass surgery. As already stated, circulating concentrations of GLP-1 obtained after gastric bypass surgery are much lower than those obtained with GLP-1 receptor agonists, although bariatric surgery is associated with a greater and more sustainable improvement to health. Above all, gastric bypass surgery promotes the secretion of endogenous GLP-1 in the digestive tract, probably the most important place for the physiological effects of GLP-1, thus restoring this hormone and its multiple modes of action [73] (Figure 2).

## 9. What Can We Conclude?

One message is becoming increasingly apparent from studies in the literature: the physiological effects of GLP-1 must not be confused with its pharmacological effects. Indeed, the effects of receptor agonists of GLP-1 should not necessarily be considered to be representative of the physiological effects of GLP-1. To explain these inconsistencies, it is important to consider the dose used. The physiological effects of GLP-1 can probably be restricted to the incretin effect, in which portal GLP-1 and the “hepatoportal sensor” play essential roles. In this case, GLP-1 acts probably as a neurotransmitter. Conversely, any pharmacological manipulation (supraphysiological) of GLP-1 involves GLP-1 receptors in peripheral tissues, which explains why vastly greater doses of GLP-1 are required to obtain a substantial effect on appetite or blood sugar levels. In this case, GLP-1 acts as a circulating hormone. An example of this concept is the work by Ahrén [43], in which mice were denervated at birth by the injection of capsaicin: at physiological doses of GLP-1, there was no incretin effect and no effect on satiety; however large doses were able to cause these two phenomena. This work demonstrates that physiological doses of GLP-1 require a functional nervous system and that pharmacological doses of GLP-1 are mediated by other pathways. Thus, the mechanism of action of GLP-1 is probably specifically determined by the dose used. This does not exclude the possibility that pharmacological compounds have other specific mechanisms of action that are still unknown and await discovery. Thus, a new field of research has opened up that extends beyond the physiological effects of GLP-1: the identification of molecular targets specific to receptor agonists of GLP-1 besides the targets of GLP-1 itself. This could help to improve our understanding of the mechanisms of action of these molecules to enable the development of new, more effective compounds.
